# Assessing the Interactive Effects of Graphene Oxide and Marine Heatwave Stressors on Estuarine Bivalves

**DOI:** 10.3390/toxics14040339

**Published:** 2026-04-17

**Authors:** Valéria Giménez, Beatriz Neves, Etelvina Figueira, Paula Marques, Adília Pires

**Affiliations:** 1 Centre for Environmental and Marine Studies (CESAM), Department of Biology, University of Aveiro, 3810-193 Aveiro, Portugal; valeriacgimenez@ua.pt (V.G.); beatrizrneves@ua.pt (B.N.); efigueira@ua.pt (E.F.); 2 Centre for Mechanical Technology and Automation (TEMA), Department of Mechanics, University of Aveiro, 3810-193 Aveiro, Portugal; paulam@ua.pt

**Keywords:** bivalves, marine heatwaves, nanomaterials, biochemical analysis, oxidative stress

## Abstract

Coastal ecosystems are increasingly threatened by climate change, especially the rising frequency of marine heatwaves (MHWs), which often co-occur with emerging nanomaterials such as graphene oxide (GO), whose ecological risks are still being evaluated. While the effects of GO have been studied in isolation, little is known about its interaction with thermal stress events. This research studied the combined effects of temperature (18 °C and 23 °C, simulating control and MHW conditions) and GO nanosheets exposure (0.01 mg/L) on two key estuarine bivalves: the clam *Scrobicularia plana* and the mussel *Mytilus galloprovincialis*. After 7 days of exposure (duration of many MHWs), energy metabolism, antioxidant defenses, oxidative damage, and neurotransmission were assessed. The results revealed that clams exhibited lower ETS and SOD activity when exposed to MHWs and lower SOD and AChE activities at MHW + GO, compared to the control treatment. Mussels relied primarily on SOD activity across treatments but showed increased susceptibility to GO nanosheets, with higher LPO levels and a significant reduction in AChE activity when exposed to GO at both temperatures. Overall, our findings suggest that *S. plana* shows a stronger response to the environmental alterations tested than *M. galloprovincialis*. Combined exposure to GO + MHW triggers species-specific biochemical responses in estuarine bivalves, highlighting how physiological traits shape the assessment of ecological risks posed by nanomaterial pollution under climate change.

## 1. Introduction

Estuarine ecosystems rank among Earth’s most productive and dynamic environments, functioning as crucial transition zones linking freshwater and marine systems [1]. They perform essential ecological functions, such as nutrient cycling, primary production, sediment stabilization, and providing nursery grounds for a wide range of species. In addition, they support fisheries and aquaculture, which are of major economic and cultural importance [2]. However, estuaries are increasingly threatened by anthropogenic stressors, including pollution, habitat modification, and climate change, making them vulnerable to disturbances that can cascade across trophic levels [3]. In addition to anthropogenic pressures, estuarine organisms are increasingly facing acute thermal anomalies, with marine heatwaves (MHWs) among the most severe manifestations of climate change in coastal areas. MHWs are characterized as events wherein the ocean temperature goes beyond an exceptional threshold for a minimum period of five consecutive days [4,5]. These events are described in the literature as arising from persistent high-pressure systems and weak winds, enhanced solar radiation, and reduced ocean heat loss, which together promote sustained warming of surface waters [6]. Although the global average duration is increasing, the study by Ren et al. (2025) [4] shows that MHWs can last from just one week up to several months, with the analysis period (1982–2022) revealing an increasing trend in duration, and are projected to intensify under future warming scenarios. These extreme events can disrupt physiological performance, feeding activity, and reproduction in marine invertebrates, with cascading impacts on ecosystem structure and services [7,8]. Along the Portuguese coast (including the Aveiro region), MHWs typically occur in spring and summer with anomalies of +2–5 °C above baseline temperatures (~18–22 °C), driven by atmospheric blocking and reduced upwelling [9]. Bivalves, in particular, are highly sensitive to elevated temperatures, often exhibiting oxidative stress, metabolic disruption, and mortality under MHW conditions [10,11]. However, studies such as that by Peruzza et al. (2023) [10] reveal that the consequences of non-lethal MHWs are often overlooked, leading to reproductive dysfunction, a significant depletion of energy reserves, and dysbiosis in the digestive gland microbiota of bivalve mollusks. Additionally, elevated temperatures can increase organisms’ sensitivity to other pollutants by altering their toxicity. Also, recent studies illustrate this synergistic effect in mussels *Mytilus coruscus* by demonstrating that co-exposure to titanium dioxide nanoparticles (TiO_2_) drastically exacerbates oxidative stress, inducing dysbiosis, increasing toxicity and physiological damage [12].

Graphene oxide (GO), a derivative of graphene characterized by a high surface area and multiple oxygen-containing functional groups, has attracted considerable attention due to its versatile applications in fields such as biomedicine, electronics, energy storage, and water purification [13,14,15,16]. Its hydrophilic nature and ability to disperse in aqueous solutions increase the likelihood of environmental release through industrial effluents and waste streams, particularly into aquatic ecosystems [17]. Emerging studies on GO ecotoxicity reveal diverse, species-dependent responses (Table 1). For instance, exposure of the marine bivalve *Crassostrea virginica* to 2.5 and 5 mg/L of GO nanomaterial for 72 h resulted in increased lipid peroxidation (LPO) and glutathione S-transferases (GSTs) activity, suggesting oxidative stress as a central toxic mechanism [18]. Also, exposure to GO induces ecotoxicological effects in *Artemia franciscana*, where significant mortality (25%) was observed only in adult individuals and exclusively at the highest concentration (100 µg/mL) after 72 h [19]. Additionally, this maximum GO concentration caused a significant and time-dependent activation of GSTs. Also, chronic GO exposure (0.2, 1, 5 mg/L for 28 days) enhanced toxicity in the freshwater clam *Corbicula flumine* through oxidative stress amplification [20]. In the polychaete *Hediste diversicolor*, GO nanosheets (0.01, 0.1, 1, and 10 mg/L GO) elicited behavioral, physiological, and biochemical alterations, including antioxidant responses [21].

Although progress has been made, a substantial knowledge gap persists regarding the combined effects of GO and climate-related stressors, such as MHWs, in estuarine contexts. Estuarine species are often exposed to both contaminant inputs and temperature fluctuations, which may act synergistically to exacerbate biochemical, physiological, and behavioral stress responses. Among the species inhabiting estuarine environments, *Mytilus galloprovincialis* (the Mediterranean mussel) and *Scrobicularia plana* (the peppery furrow shell) hold ecological and economic importance. *M. galloprovincialis* is a filter-feeding bivalve widely distributed and cultivated along European coasts, valued for its role in water filtration and as a commercial species [22]. Meanwhile, *S. plana* inhabits intertidal mudflats and contributes significantly to sediment bioturbation, influencing nutrient dynamics and acting as prey for higher trophic levels [23]. These complementary ecological roles make both species excellent bioindicators for ecotoxicological assessments, providing valuable insights into environmental health and the impact of pollutants [24,25]. Since *M. galloprovincialis* and *S. plana* play key ecological roles in filtration and nutrient cycling, and are widely used as sentinel species, understanding how GO and MHWs interact to affect their physiological and biochemical performance is crucial. To evaluate the subcellular effects of GO and MHW in estuarine bivalves, we used an integrative battery of biochemical biomarkers to assess energy metabolism, oxidative stress, oxidative damage, and neurotoxicity. Electron transport system (ETS) activity and total protein (PROT) content were used as proxies for cellular energy metabolism and basal energy reserves, as changes in ETS and energy stores are widely applied to assess alterations in metabolic capacity and physiological condition in marine bivalves and other marine invertebrates [26,27,28]. Superoxide dismutase (SOD) and GSTs were selected as key antioxidant and phase II detoxification enzymes; LPO and protein carbonylation (PC) were determined as markers of oxidative damage to lipids and proteins, respectively, which are among the most sensitive and commonly used endpoints of contaminant-induced oxidative stress in clams, mussels, and other invertebrates [18,20,29,30,31,32,33]. In addition, acetylcholinesterase (AChE) activity was included as a neurotoxicity biomarker, since AChE inhibition in bivalves is a well-established indicator of exposure to neurotoxic contaminants and altered neuronal function in coastal environments [31,34,35,36] (Table 1).

This study, therefore, aims to investigate how GO exposure, alone and in combination with simulated MHW conditions, modulates physiological and biochemical biomarkers in *M. galloprovincialis* and *S. plana*. These findings will help clarify the risks posed by emerging nanomaterials under future climate scenarios in coastal ecosystems.

**Table 1:** Summary of experimental ecotoxicology studies evaluating graphene oxide (GO) exposure in aquatic invertebrate species. GLY: glycogen; ETS: electron transport system; LPO: lipid peroxidation; CAT: catalase; SOD: superoxide dismutase; GSTs: glutathione S-transferases; GSH/GSSG: reduced (GSH) and oxidized (GSSG) glutathione content; PROT: protein content; LIP: lipids; PC: protein carbonylation; IDH: isocitrate dehydrogenase; GPx: glutathione peroxidase; AChE: acetylcholinesterase.

Species	Matherial and Size	Concentration in Water (W)/Sediment (S)	Time of Exposure	Endpoints	Authors
*Tubifex tubifex*	GO nanoparticles (150 nm)	20 or 180 μg GO/g dw (S)	5 days	Mortality, avoidance, burrowing behavior	Zhang et al., 2017 [15]
*Danio rerio*	GO nanosheets (0.3 to 2.6 μm)	0.1, 0.01, and 0.001 mg/L (W)	96 h	Hatching rate, heart rate, malformation, mortality, organ development, gene expression and oxidative damage	Zhang et al., 2017 [16]
*Crassostrea virginica*	GO (300–700 nm)	0, 2.5 and 5 mg/L (W)	14 days	Exposure water assessments for GO size and concentration, LPO, GSTs and total protein	Khan et al., 2019 [18]
*Artemia franciscana*	GO (6000 and 30,000 nm)	0, 1, 10 and 100 μg/mL (W)	21 days	Mortality, genetic identification, ROS detection, Enzyme extraction (GSTs, AChE, quantification of enzymatic activity), protein content, uptake of GO	Cavion et al., 2020 [19]
*Corbicula fluminea*	GO sheets (300–1000 nm)	0, 0.2, 1, 5 mg/L (W)	28 days	Filtration rate, Histopathological analysis, reactive oxygen species and antioxidant entities, transcriptional profiling of genes through qRT-PCR, enhanced integrated biomarker response (EIBR) analysis	Bi et al., 2022 [20]
*Hediste diversicolor*	GO nanosheets (∼790 nm)	0, 0.01, 0.1, 1 and 10 mg/L (W)	28 days	Mortality, regenerative capacity, mucus production, feeding activity, burrowing assay, ETS, GLY, LIP, PROT, SOD, GSTs, LPO, PC,	Pires et al., 2022 [21]
*Diopatra neapolitana*	GO (200–400 nm)	0.01, 0.10 and 1.00 mg/L (W)	28 days	Mortality, regenerative capacity, GLY, ETS, LPO, CAT, SOD, GSTs, GSH/GSSG	De Marchi et al., 2017 [27]
*Ruditapes philippinarum*	GO nanosheets	0, 0.01, 0.1 and 1.0 mg/L	28 days	PROT, LPO, SOD, GSH, CAT, GSTs	Britto et al., 2021 [28]
*Mytilus galloprovincialis*	GO nanosheets (0.1 to 0.3 μm)	0.5 mg/L (W)	7 days	Antioxidant response to GO (CAT, GSTs, SOD, GSH), cytotoxicity, effects on gene expression, alterations in the cellular ultrastructure and uptake	Meng et al., 2020 [32]
*Artemia salina*	GO (1- 5 μm)	0, 1, 10 and 50 mg/L (W)	48 h	Microscope observation of GO uptake, LPO, SOD	Lu et al., 2018 [33]
*Hediste diversicolor* and *Arenicola marina*	GO nanosheets (∼790 nm)	0, 0.001, 0.01, 0.1, 1, 10 mg GO/Kg dw (W)	24 h	Avoidance assay, sediment particle reworking activity using fluorescent sediment profile imaging, post-exposure burrowing activity, PROT, AChE, SOD, LPO	Figueiredo et al., 2024 [35]
*Mytilus galloprovincialis*	GO nanoplatelets (500 nm)	500 μg/L (W)	7 days	CAT, AChE, IDH, GSTs, GPx, gamete development, gonad index, histopathology of mussel gonad and of digestive gland, determination of graphene oxide in mussel tissues and feces, cellular biomarkers in hemocytes	González-Soto et al., 2023 [36]

## 2. Materials and Methods

### 2.1. Graphene Oxide Nanosheets

A commercial aqueous dispersion of graphene oxide (GO) nanosheets (0.4 wt %) was purchased from Graphenea (San Sebastian, Spain). The supplier reports that the lateral dimensions of the GO nanosheets were highly variable, with a mean flake size of approximately 790 nm and always below 10 μm. These specifications were previously validated by atomic force microscopy (AFM), which confirmed the material’s characteristic sheet-like morphology. AFM analyses further revealed that more than 95% of the flakes consist of monolayers exhibiting a thickness of ~0.97 nm, while the remainder are few-layered nanosheets [37].

### 2.2. Test Organisms and Acclimation

Mussels (*Mytilus galloprovincialis*) and clams (*Scrobicularia plana*) were manually collected from a reference site in Ria de Aveiro Lagoon, Portugal (40°38′39″ N 8°44′06″ W and 40°36′59″ N 8°44′22″ W, respectively) previously characterized as relatively unimpacted in earlier studies (low trace metals, PAHs, and PCBs) in baseline studies [38,39]. Organisms were brought live to the lab and acclimated in glass aquaria: mussels in artificial seawater, and clams in artificial seawater with sediment (water: sediment ratio of 4:1). The artificial seawater was at 28 salinity, and organisms were maintained at a controlled temperature (18 °C). During acclimation, clams and mussels were fed a liquid phytoplankton replacement, Phytonic (Tropic Marine), at a daily rate of 0.13 mL per 25 L of artificial water, to maintain their nutritional condition. For each species, individuals of similar size were selected to obtain a homogeneous sample (*S. plana*: length 21 ± 3 mm, width 16 ± 2 mm; *M. galloprovincialis*: length 54 ± 0.6 mm, width 37.8 ± 0.8 mm).

### 2.3. Experimental Design

**Figure 1:**
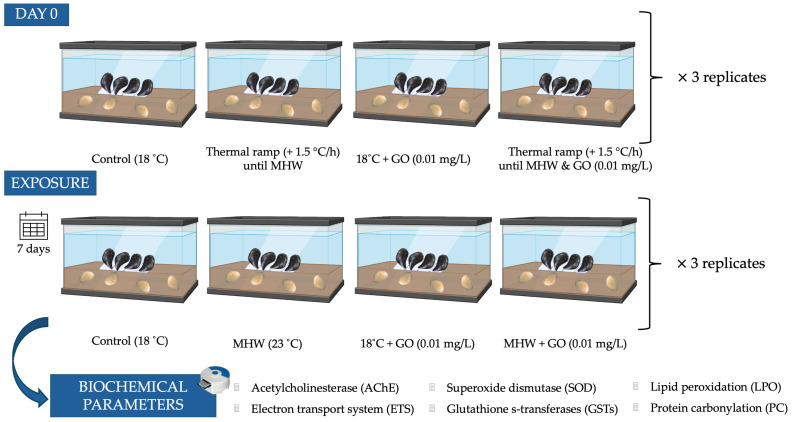
Schematic representation of the experimental design used to evaluate the combined effects of marine heatwaves (MHW) and graphene oxide (GO) on the bivalves *Scrobicularia plana* (buried in marine sediment; infaunal) and *Mytilus galloprovincialis* (exposed in the water column; epifaunal). Organisms were exposed for 7 days under four conditions: control (18 °C), MHW (23 °C), GO exposure (0.01 mg L^−1^) at 18 °C, and combined MHW and GO exposure. Each treatment was performed in triplicate aquaria containing four individuals of each species.

After a two-week acclimation period, 48 individuals of each species (3 replicates × 4 organisms per replicate × 4 conditions) were used. Clams and mussels were exposed to different environmental conditions to assess the combined effects of temperature and graphene oxide (GO) for 7 days (Figure 1). Both species were exposed simultaneously in the same aquaria, but each was maintained under conditions that reflect its natural ecological niche (infaunal vs. epifaunal). Clams and mussels were exposed in tanks containing a layer of natural beach sand (marine sediment; clean fine sand; 1.53% fines; 0.35% total organic matter) collected from the reference site, previously sieved and rinsed to remove debris. Each treatment had three aquaria, each with a total of four individuals from each species allocated to one of the following four treatments: (i) 18 °C (control), (ii) marine heatwave (MHW) (23 °C), (iii) 18 °C with GO (0.01 mg/L) exposure, and (iv) MHW with GO exposure (0.01 mg/L). The control temperature of 18 °C was defined based on the current average sea surface temperature in the study area (16–19 °C; IPMA, 2025 [40]). The highest temperature of 23 °C (+5 °C above the control) was used to simulate end-of-century global warming scenarios corresponding to an increase of approximately 4–7 °C (IPCC, 2023 [41]). At the beginning of the exposure, bivalves were subjected to a thermal ramp to reach the experimental temperature of 23 °C by increasing the water temperature 1.5 °C per hour [42]. An exposure concentration of 0.01 mg/L GO was selected, based on predicted environmentally relevant concentrations for GO in aqueous systems, which range from 1 μg/L to 1 mg/L [16,43]. Water renewal was performed every 2 days, and during each renewal, the contaminant (GO) was re-added to the appropriate tanks to maintain exposure conditions. For tanks simulating heatwave conditions (23 °C), replaced water was pre-heated to ensure consistent thermal exposure. At the end of the exposure, bivalves were collected and frozen at −80 °C for biochemical analysis.

### 2.4. Biochemical Analysis

For biochemical analysis, frozen specimens (3 per aquarium, 9 per condition) were homogenized with a mortar and a pestle in liquid nitrogen. Homogenates of the whole soft tissues were obtained to provide an integrated measure of organism-level responses and overall physiological status, and to ensure sufficient material to run the full biomarker battery in both species. This approach is frequently employed in bivalve biomonitoring to reflect cumulative stressor effects on the entire organism, despite lacking organ-specific resolution [44,45,46]. Then, samples were sonicated in 0.1 M phosphate buffer, pH 7.4, using an ultrasound tissue disrupter (Sonics Vibra Cell VCX 130, SONICS, Newtown, CT, USA). Homogenates were divided into three aliquots: one for determination of lipid peroxidation (LPO); another where the homogenate was centrifuged for 3 min, at 3300× *g*, at 4 °C to determine cholinesterase activity (ChE) and electron transport system (ETS); and another that was centrifuged at 10,000× *g*, at 4 °C for 20 min, to determine protein content (PROT), superoxide dismutase (SOD), glutathione S-transferases (GSTs), and protein carbonylation (PC). All absorbances were read using a MicroDigital Mobi Microplate Spectrophotometer (Emin, Seoul, Republic of Korea).

#### 2.4.1. Energy Parameters

Electron transport system (ETS) activity was determined according to the method described by King and Packard (1975) [47], with modifications introduced by De Coen and Janssen (1997) [48]. Absorbance was measured at 490 nm every 25 s over a total period of 10 min. The amount of formazan produced was calculated using a molar extinction coefficient of ε = 15,900 M^−1^ cm^−1^, and results were presented in nmol min^−1^ per g FW.

Protein content (PROT) was quantified using the Biuret method [49]. Bovine serum albumin (BSA) from Sigma-Aldrich (Saint Louis, MO, USA) served as the standard, with a concentration range of 0–40 mg mL^−1^. Absorbance readings were taken at 540 nm. Protein content results were expressed as mg g^−1^ of fresh weight (FW).

#### 2.4.2. Antioxidant Enzymes

Glutathione S-transferases (GSTs) activity was assessed following the protocol described by Habig et al. (1974) [50]. Absorbance readings were taken every 15 s over a 5 min period, monitoring the increase in absorbance at 340 nm. Enzyme activity was calculated using a molar extinction coefficient of ε = 9.6 mM^−1^ cm^−1^ and expressed as U g^−1^ FW.

Superoxide dismutase (SOD) activity was evaluated using the method described by Beauchamp (1971) [51]. Enzyme activity was measured at 560 nm following incubation. Results were reported in U g^−1^ FW.

#### 2.4.3. Oxidative Damage Parameters

Lipid peroxidation (LPO) was assessed using the method described by Buege and Aust (1978) [52]. The analysis involved quantifying malondialdehyde (MDA) concentration using a molar extinction coefficient of ε = 1.56 × 10^5^ M^−1^ cm^−1^. Results were reported as nmol of MDA per g of FW.

Protein carbonylation (PC) levels were determined following the protocol outlined by Mesquita et al. (2014) [53], with modifications introduced by Udenigwe et al. (2016) [54]. Carbonyl content was calculated using ε = 22,308 mM^−1^ cm^−1^ and expressed as μmol g^−1^ FW.

#### 2.4.4. Acetylcholinesterase Enzymatic Activity

Acetylcholinesterase (AChE) was evaluated according to Ellman et al. (1961) [55], incorporating the modifications described by Mennillo et al. (2017) [56]. Acetylthiocholine iodide served as the substrate for quantification. The degradation rate of acetylthiocholine was monitored continuously for 5 min at 412 nm. Specific activity was corrected for substrate spontaneous hydrolysis and expressed as nmol per min per g FW, using ε = 13,600 nM^−1^ cm^−1^.

### 2.5. Statistical Analysis

Biochemical descriptors (PROT content, AChE, ETS, SOD, GSTs, LPO, and PC) were analyzed through permutational multivariate analysis of variance using the PERMANOVA+ add-on in PRIMER v6 [57]. For each descriptor, significant differences among treatments were subsequently evaluated. The permutation procedure consisted of unrestricted permutations of the raw data, with a maximum of 9999 iterations. Pairwise comparisons between experimental conditions were assessed using the Monte Carlo test to obtain specific numerical outcomes. Statistical significance was established at *p* < 0.05. All descriptors were analyzed following a one-way hierarchical design. The null hypotheses that were tested were (a) for each biomarker, and for each species, no significant differences were expected among treatments (CTL 18 °C, 18 °C + 0.01 mg GO/L, CTL MHW and MHW + 0.01 mg GO/L) within the same species (identified in figures with uppercase letters for *M. galloprovincialis* and lowercase letters for *S. plana*) and (b) between species, no significant differences were expected between the same treatment (identified in figures with an asterisk).

Principal Coordinate Ordination Analysis (PCO) was used to visualize the biochemical results for each organism with each treatment. The biochemical markers that were highly correlated (*r* > 0.75) were represented as superimposed vectors in the PCO graph.

## 3. Results

### 3.1. Energy-Related Parameters

**Figure 2:**
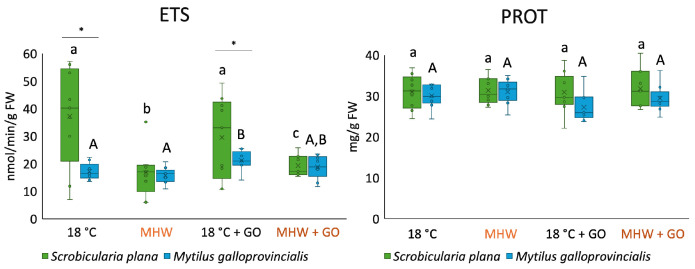
Electron transport system (ETS) activity and protein (PROT) content in *Scrobicularia plana* and *Mytilus galloprovincialis* exposed to control and graphene oxide (GO) nanosheet treatments at 18 °C and MHW (23 °C). Data are presented as boxplots showing median, interquartile range, and outliers. Within each species, lowercase letters (a, b, c) indicate statistically significant differences among treatments in *S. plana* (*p* < 0.05), uppercase letters (A, B) indicate significant differences among treatments in *M. galloprovincialis*. Asterisks (*) denote significant interspecific differences under the same treatment (*p* < 0.05).

ETS activity differed significantly between species, temperature scenarios, and GO treatments (Figure 2). In *S. plana*, exposure to MHW, both alone and combined with GO, led to a significant decrease in ETS activity compared to the control condition (18 °C). In *M. galloprovincialis*, ETS activity was significantly higher at 18 °C + GO. At 18 °C and 18 °C + GO conditions, *S. plana* had significantly higher levels of ETS activity compared to *M. galloprovincialis*.

Regarding protein levels, for each species and among species, no significant differences were observed among treatments (Figure 2).

### 3.2. Antioxidant Defenses

**Figure 3:**
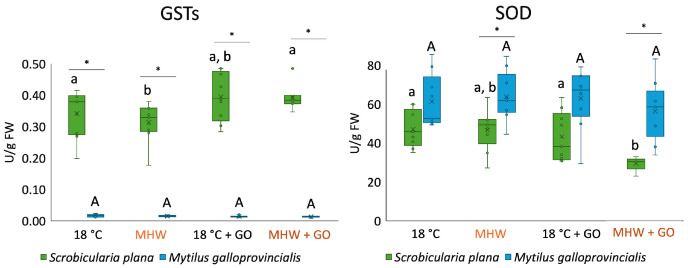
Glutathione S-transferases (GSTs) and superoxide dismutase (SOD) activities in *Scrobicularia plana* and *Mytilus galloprovincialis* exposed to control and graphene oxide (GO) nanosheet treatments at 18 °C and MHW (23 °C). Data are presented as boxplots showing median, interquartile range, and outliers. Within each species, lowercase letters (a, b) indicate statistically significant differences among treatments in *S. plana* (*p* < 0.05), uppercase letters (A) indicate significant differences among treatments in *M. galloprovincialis*. Asterisks (*) denote significant interspecific differences under the same treatment (*p* < 0.05).

GST activity showed strong species-specific differences (Figure 3). In *S. plana,* GST activity significantly decreased under MHW exposure. In *M. galloprovincialis,* no significant differences were detected among treatments. GST activity significantly increased in all treatments in *S. plana* when compared with *M. galloprovincialis*.

Concerning SOD activity in *S. plana*, enzyme activity significantly decreased at MHW + GO (Figure 3). In *M. galloprovincialis*, although values fluctuated among treatments, no significant differences were detected. SOD activity in *M. galloprovincialis* was significantly higher than in *S. plana* under exposure to MHW and MHW + GO.

### 3.3. Oxidative Damage

**Figure 4:**
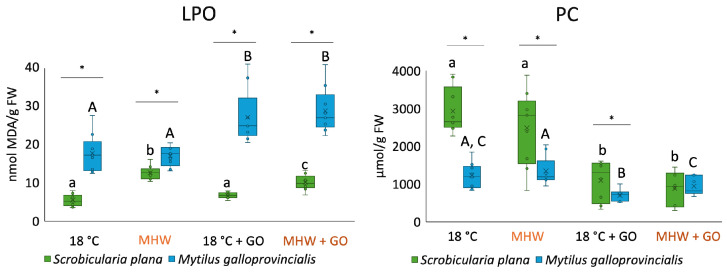
Lipid peroxidation (LPO) and protein carbonylation (PC) levels in *Scrobicularia plana* and *Mytilus galloprovincialis* exposed to control and graphene oxide (GO) nanosheet treatments at 18 °C and MHW (23 °C). Data are presented as boxplots showing median, interquartile range, and outliers. Within each species, lowercase letters (a, b, c) indicate statistically significant differences among treatments in *S. plana* (*p* < 0.05), uppercase letters (A, B, C) indicate significant differences among treatments in *M. galloprovincialis*. Asterisks (*) denote significant interspecific differences under the same treatment (*p* < 0.05).

LPO levels significantly increased in *S. plana* exposed to MHW and MHW + GO treatments compared to the control (Figure 4). In *M. galloprovincialis*, LPO values increased significantly when organisms were exposed to GO nanosheets. Among species, *M. galloprovinciallis* had significantly higher LPO levels compared to *S. plana.*

In *S. plana*, PC significantly decreased in treatments with GO (18 °C + GO and MHW + GO). In *M. galloprovincialis* at 18 °C + GO, PC levels significantly decreased compared to the control (Figure 4). In MHW + GO, PC levels were significantly lower compared to only MHW exposure. Concerning PC-level comparison among species, there were significant differences between 18 °C, MHW, and 18 °C + GO treatments, with *S. plana* having the highest PC.

### 3.4. Acetylcholinesterase Activity

**Figure 5:**
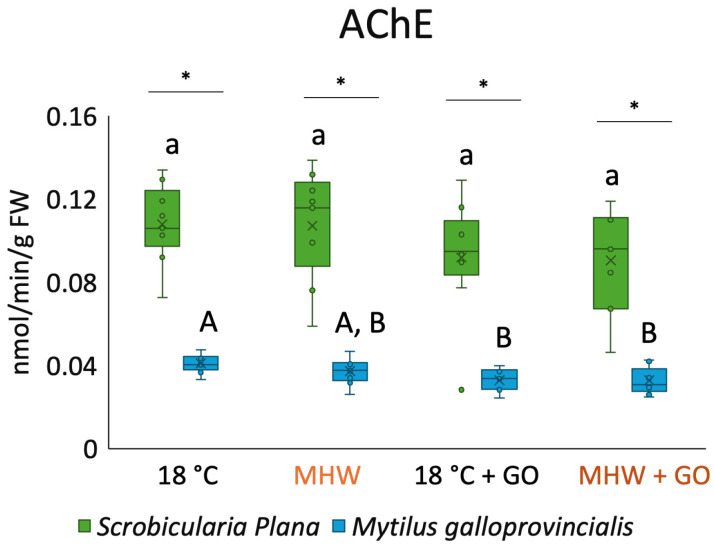
Acetylcholinesterase (AChE) activity in *Scrobicularia plana* and *Mytilus galloprovincialis* exposed to control and graphene oxide (GO) nanosheet treatments at 18 °C and MHW (23 °C). Data are presented as boxplots showing median, interquartile range, and outliers. Within each species, lowercase letters (a) indicate statistically significant differences among treatments in *S. plana* (*p* < 0.05), uppercase letters (A, B) indicate significant differences among treatments in *M. galloprovincialis*. Asterisks (*) denote significant interspecific differences under the same treatment (*p* < 0.05).

Regarding AChE activity for *S. plana,* no significant differences were detected among treatments (Figure 5). For *M. galloprovincialis*, AChE activity decreased significantly in treatments with GO nanosheets. All treatments showed significant differences among species, with *M. galloprovincialis* exhibiting the lowest overall AChE activity.

### 3.5. Multivariate Analysis

**Figure 6:**
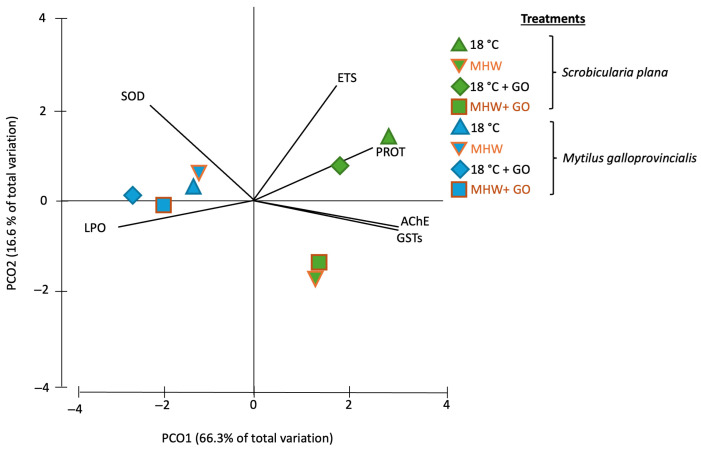
Principal Coordinate Ordination Analysis (PCO) based on all biomarker responses in *Scrobicularia plana* (green) and *Mytilus galloprovincialis* (blue) exposed to control and graphene oxide (GO) nanosheet treatments at 18 °C and MHW (23 °C) (*r* > 0.75). Treatments are coded as: 18 °C (control), MHW (MHW control), 18 °C + GO, and MHW + GO. Vectors indicate the contribution of each biomarker to the ordination.

A Principal Coordinate Ordination Analysis (PCO) was conducted based on the results of the biochemical markers analyzed in *S. plana* and *M. galloprovincialis* exposed to different treatments (18 °C, MHW, 18 °C + GO, MHW + GO) (Figure 6). PCO explained 82.9% of the total variation in the dataset, with PCO1 explaining 66.3% of the total variation and PCO2 16.6%.

Regarding PCO1, a clear interspecific separation was observed, with *S. plana* exposed to the four conditions tested clustering on the positive side, while *M. galloprovincialis* grouped on the negative side. On the positive side of PCO1, *S. plana* individuals from the 18 °C and 18 °C + GO groups were closely associated with PROT content (correlation of 0.80), and organisms exposed to MHW and MHW + GO were more associated with GSTs and AChE activities (correlation of 0.95 and 0.97, respectively). On the negative side of PCO1, *M. galloprovincialis* exposed to all conditions showed a moderate association with SOD activity and a high association with LPO levels (correlation of −0.71 and −0.96, respectively) (Table S1).

Concerning the PCO2 axis, samples of *S. plana* at 18 °C (with and without GO) were positioned on the positive side, being more associated with higher ETS activity (correlation of 0.73) (Figure 6, Table S1). On the other hand, *S. plana* individuals under MHW and MHW + GO were on the negative side of the axis. *M. galloprovincialis* exhibited minimal dispersion along PCO2, clustering proximally to the origin.

## 4. Discussion

The present study demonstrated that exposure to MHW and graphene oxide (GO) nanosheets elicits distinct biochemical and physiological responses in the two bivalves examined, the clam *Scrobicularia plana* and the mussel *Mytilus galloprovincialis*. These findings reveal that the interaction between thermal stress and nanomaterial contamination does not follow a uniform pattern across taxa, but instead reflects species-specific sensitivities shaped by their ecological niches and physiological traits [18,58].

After the experimental period, multivariate analysis showed that PCO explained more than 80% of the total variation, and temperature emerged as a major driver of the biochemical responses, particularly in *S. plana*. In clams, samples were separated according to temperature treatments, with those exposed to 18 °C associated mainly with ETS and PROT, while samples exposed to MHW treatments were mainly correlated with AChE and GST activities, suggesting a higher sensitivity of *S. plana* to thermal stress. Specifically, the MHW condition alone led to a decrease in GST activity, suggesting a temperature-induced compromised detoxification capacity. In contrast, *Mytilus galloprovincialis* samples clustered more tightly across temperatures, with all treatments associated with SOD and LPO, suggesting that the tested MHW scenario induced less pronounced temperature-specific separation in mussels and that oxidative endpoints were responsive under both thermal conditions.

In *S. plana*, MHW exposure significantly reduced ETS activity compared to treatments at 18 °C. ETS represents mitochondrial respiratory capacity and is a key proxy for metabolic performance in marine bivalves [59]. Increased or decreased ETS activity may indicate shifts in energy allocation in response to changing environmental stressors such as temperature or pollutants [26]. The decrease in ETS activity observed in these clams under MHW exposure is consistent with a metabolic rate depression-like response. This strategy, documented in other organisms, including bivalves such as *Sphaerium occidentale* and *Musculinum securis*, consists of reducing metabolic rate to cope with exposure to a stressor [60,61]. This suggests that, under an MHW scenario, *S. plana* may adjust its metabolic profile by reducing ETS activity and modulating other physiological responses, including antioxidant defenses [62,63]. Thermal stress also affected the antioxidant and detoxification capacities in clams. GST activity decreased under MHW, whereas in *M. galloprovincialis,* GSTs did not differ significantly among treatments. Glutathione-S-transferases (GSTs) are a group of detoxification enzymes that catalyze the conjugation of reduced glutathione (GSH) to xenobiotic substrates for subsequent elimination from the organism [64], and the lack of response in mussels suggests that mussels may rely more heavily on first-line antioxidant enzymes such as SOD, which dismutates superoxide radicals, rather than GST-dependent detoxification pathways, which target secondary oxidative products and xenobiotics [65]. Although SOD activity in *M. galloprovincialis* was higher than in *S. plana*, mussels did not exhibit significant differences among treatments. These higher baseline SOD levels in mussels may reflect an inherent physiological capacity shaped by daily environmental fluctuations characteristic of intertidal habitats [66], potentially masking the effects of MHW. The combination of higher SOD and lower GST activity in mussels suggests that efficient SOD-mediated superoxide dismutation likely contributed to lower ROS burden, potentially reducing the demand for downstream GST-dependent detoxification of secondary oxidative products, consistent with patterns where SOD upregulation precedes GST modulation in bivalves under contaminant stress [67]. At the same time, LPO levels increased significantly under MHW (with and without GO), indicating enhanced membrane oxidative damage and reinforcing the role of temperature as a key driver of oxidative stress in *S. plana*. Given its benthic, infaunal lifestyle and the typically more stable thermal regime inside sediments, these results are consistent with a narrower thermal tolerance window and a higher vulnerability to abrupt warming events. In contrast, *M. galloprovincialis* maintained ETS activity relatively stable across treatments (except for an increase at 18 °C + GO), suggesting that the mussels did not undergo the same degree of temperature-induced metabolic depression observed in clams. This pattern is consistent with the broader thermal tolerance and higher plasticity described for intertidal mussels, which routinely experience daily temperature fluctuations and episodic heat events in their natural habitat [68]. The predominance of SOD and LPO in the multivariate patterns across all mussel treatments indicates that oxidative processes were responsive, but alterations in temperature within the tested range acted as a more moderate driver compared to their effect on *S. plana*.

Graphene oxide exposure mainly affected contaminant-driven endpoints, particularly in *M. galloprovincialis*. Mussels exposed to GO showed increased LPO, suggesting that GO could enhance reactive oxygen species (ROS) production and thereby promote lipid peroxidation [69]. Similar increases in LPO have been reported in *C. virginica* exposed to GO [18], and in *M. galloprovincialis* exposed to metals like Cu, Cd, Pb, and Fe during six days [69], as well as to benzo(a)pyrene and Cu [70], showing the sensitivity of this species to contaminant exposures. Mussels also showed an inhibition of AChE activity. AChE is a serine hydrolase that catalyzes the hydrolysis of acetylcholine into acetate and choline, terminating cholinergic neurotransmission at synapses and neuromuscular junctions [71]. In this study, no significant differences between treatments were observed in clams, in contrast to mussels, which exhibited decreased AChE activity in the GO treatments, with a significant decrease at 18 °C + GO. These findings indicate that GO exerts a neurotoxic effect in mussels, potentially through interactions with the enzyme’s active site, forming a GO-AChE complex, reducing the activity of the enzyme [72]. Moreover, although AChE activity was reduced under GO exposure to both temperatures, the significantly lower values observed in the 18 °C + GO treatment may be related to the thermal preference of *M. galloprovincialis* (between 17 °C and 20 °C) [73]. At this temperature, mussels are within their optimal physiological range and likely maintain normal filtration activity, thereby increasing their uptake of GO particles [68]. This enhanced accumulation may have intensified the neurotoxic effects of GO, leading to the pronounced AChE inhibition observed at 18 °C. This inhibition is ecologically relevant, as acetylcholine accumulation can impair neuromuscular function and compromise vital functions such as filtration, feeding, and mobility [74]. In clams, GO alone induced more modest biochemical changes, with limited effects on ETS and protein content and no marked neurotoxicity, suggesting that *S. plana* is comparatively less sensitive to GO than to thermal stress under the present conditions. However, it is important to note that *S. plana* was exposed in the presence of natural sediment, which may influence GO distribution, aggregation, and bioavailability, potentially modulating the magnitude of biomarker responses observed in this species. The increase in ETS activity observed in mussels at 18 °C + GO suggests that, at the species’ thermal optimum, normal valve opening and filtration may have favored GO uptake. Although not directly assessed in this study, temperature-induced changes in GO aggregation may potentially influence its bioavailability and the effective dose reaching the organisms [75], which could contribute to the observed alterations in ETS activity, as nanoparticle aggregation behavior is known to model exposure in toxicity in aquatic systems [76], leading to a higher internal dose and a stimulation of metabolic capacity to sustain detoxification and repair. GO exposure was associated with reduced PC levels in both species. PC forms via oxidation of amino acid residues, serving as stable markers to detect protein oxidative damage [77,78]. Protein carbonyls are generally considered relatively stable markers of cumulative oxidative protein damage and often increase when oxidative stress is sustained, and cellular quality-control systems become overwhelmed, which is why they are widely used as indicators of long-term impacts [79,80]. However, mildly oxidized proteins can also be efficiently removed by proteolytic pathways on short time scales, and decreases in PC have been reported under specific temperature regimes, including long-term thermal exposure in *Trematomus bernacchii* [79]. The PC decrease observed here, together with maintained or stimulated ETS and antioxidant activities, therefore likely reflects effective prevention of degradation-resistant protein damage under the present exposure conditions, rather than a complete absence of oxidative challenge [80,81]. Overall, the biomarker responses indicate that GO acts primarily as an oxidation promoter and a neurotoxic agent in *M. galloprovincialis*, whereas its effects on *S. plana* are more subtle and largely overshadowed by temperature.

Under combined MHW + GO exposure, both species exhibited signatures of multiple stressor interactions, but with contrasting dominant drivers. In *S. plana*, MHW alone and MHW + GO increased LPO, indicating that elevated temperature is a key driver of oxidative damage, with GO potentially contributing under combined stress. Although studies addressing temperature-induced oxidative responses in *S. plana* are scarce, Amorim et al. (2020) [82] reported increased LPO levels after 25 days of exposure to a temperature rise from 17 °C to 25 °C. The pronounced thermal sensitivity of clams likely reflects their deep-burrowing lifestyle; by residing within the sediment, these organisms experience more stable thermal regimes, potentially resulting in a narrower thermal tolerance window [83]. When comparing species, LPO levels in *M. galloprovincialis* were consistently higher than those in *S. plana* across all treatments, indicating inherently higher basal levels in mussels, possibly reflecting differences in membrane lipid composition and fatty acid profiles [84]. Moreover, *M. galloprovincialis* exhibited increased LPO when exposed to gadolinium (Gd) at 22 °C for 28 days [46], further highlighting the susceptibility of membrane lipids to oxidative degradation under combined stressors. In *M. galloprovincialis*, GO remained the main driver under combined exposure. LPO and AChE inhibition were most pronounced in GO treatments at both temperatures, while ETS and PROT content remained stable, indicating contaminant-driven oxidative and neurotoxic effects rather than an energetic collapse. The fact that AChE inhibition was strongest at 18 °C + GO is consistent with mussels operating within their optimal thermal range and maintaining active filtration, thereby increasing the effective GO dose reaching the nervous system, possibly affecting the neuromuscular system. The increase in LPO under MHW and/or GO exposure might indicate that membrane lipids were highly susceptible to peroxidation under thermal and contaminant stress. Overall, the biomarker patterns indicate that *S. plana* is more vulnerable to short-term thermal anomalies, whereas *M. galloprovincialis* is more sensitive to contaminant stress. Clams exhibited decreased ETS and GSTs and increased LPO under MHW, consistent with reduced metabolic capacity and impaired detoxification, which may compromise burrowing, feeding, and predator avoidance. Mussels, by contrast, showed neurotoxic and oxidative responses driven by GO (AChE inhibition and LPO increase), suggesting that chronic exposure to emerging nanomaterials may pose a greater risk to this intertidal filter feeder. These species-specific vulnerabilities highlight the importance of considering both climate-related and contaminant-related drivers when assessing the resilience of estuarine communities to marine heatwaves and nanomaterial pollution.

## 5. Conclusions

**Figure 7:**
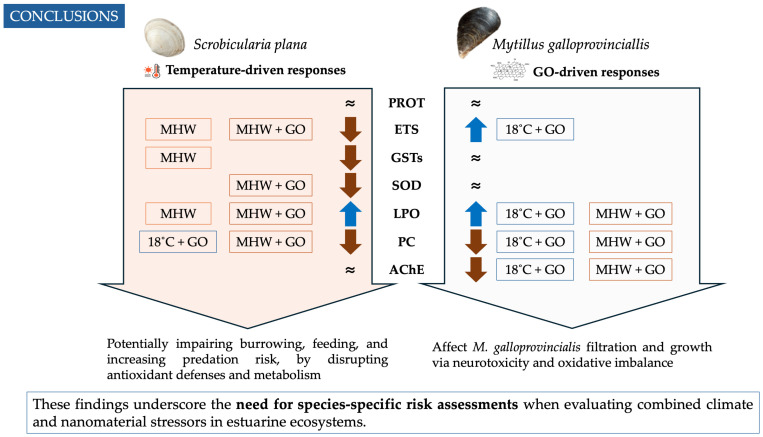
Schematic summary of the main biochemical responses to marine heatwave (MHW) and graphene oxide (GO) exposure in *Scrobicularia plana* and *Mytilus galloprovincialis*. Arrows indicate the direction of change relative to control conditions (↑ increase, ↓ decrease, ≈ no significant change). MHW denotes marine heatwave conditions (23 °C). Boxes indicate the treatments in which significant effects were observed. The diagram highlights species-specific physiological responses and their potential ecological implications. PROT: protein content; ETS: electron transport system; GSTs: glutathione S-transferases; SOD: superoxide dismutase; LPO: lipid peroxidation; PC: protein carbonylation; and AChE: acetylcholinesterase.

Overall, the PCO reinforced species-specific biochemical strategies identified in the univariate analyses, highlighting temperature-driven responses in *S. plana* and contaminant-driven effects in *M. galloprovincialis*. These differences, with *S. plana* more susceptible to thermal shifts and *M. galloprovincialis* more sensitive to GO, may compromise ecological resilience by affecting antioxidant capacity, oxidative damage, and neurotoxicity under combined MHWs and nanomaterials (Figure 7). Importantly, 23 °C represents moderate summer lagoon conditions in Ria de Aveiro, yet it elicited acute stress responses distinct from routine acclimation. This confirms that even temperature increases within the natural seasonal range can act as MHW-like stressors for *S. plana*, disrupting antioxidant defenses and metabolism, potentially impairing burrowing and feeding, and increasing predation risk, while chronic GO exposure might affect *M. galloprovincialis* filtration and growth via neurotoxicity and oxidative imbalance (Figure 7). These differential responses reflect contrasting stressor dynamics: short-term MHWs (as experimentally simulated) have a greater impact on *S. plana*, increasing its vulnerability to extreme weather events, but due to the transient nature of these events, organisms can recover and remain stress-free for extended periods until the next climatic event. If the frequency of these events is low, no strong impacts are expected on *S. plana* populations nor on the functions they play in the ecosystem. GO contamination has a higher impact on *M. galloprovincialis*, and its persistent nature can induce chronic exposure, with the potential to have more impact on *M. galloprovincialis* populations and on the ecological roles they provide. Although GO is still considered an emerging contaminant, its biological effects warrant attention, especially under combined stressor scenarios. These results emphasize the necessity for species-specific risk assessments when evaluating combined climate and nanomaterial stressors in estuarine ecosystems.

## Data Availability

The raw data supporting the conclusions of this article will be made available by the authors on request.

## Supplementary Materials

The following supporting information can be downloaded at: https://www.mdpi.com/article/10.3390/toxics14040339/s1, Table S1: Correlation coefficients between biochemical biomarkers (AChE, ETS, GSTs, LPO, PC, PROT, SOD) measured in *Scrobicularia plana* and *Mytilus galloprovincialis*, and their loadings on principal coordinate analysis (PCO1–PCO7) axes derived from the PCO analysis explaining >80% of total variation.
