# Supplementary material for: Assessing the Interactive Effects of Graphene Oxide and Marine Heatwave Stressors on Estuarine Bivalves
Source: Toxics. 2026 Apr 17;14(4):339. doi: 10.3390/toxics14040339 (PMC13120203; doi:10.3390/toxics14040339)
Supplement: Supplementary file 1 [file toxics-14-00339-s001.zip › toxics-4172695-supplementary.pdf]

Supporting information for:

Assessing the Interactive Effects of Graphene Oxide and Marine Heatwave Stressors on Estuarine Bivalves

Valéria Giménez [1], Beatriz Neves, [1] Etelvina Figueira [1], Paula A.A.P. Marques [2] and Adília Pires [1,*]

[1]Center for Environmental and Marine Studies (CESAM), Department of Biology, University of Aveiro, 3810-193 Aveiro, Portugal; valeriacgimenez@ua.pt (V.G); beatrizrneves@ua.pt; (B.N.); efigueira@ua.pt (E.F.); adilia@ua.pt (A.P.)

[2] Center for Mechanical Technology and Automation (TEMA) & Department of Mechanics, University of Aveiro, 3810-193, Aveiro, Portugal: paulam@ua.pt

*Correspondence: adilia@ua.pt

Table S1. Correlation coefficients between biochemical biomarkers (AChE, ETS, GSTs, LPO, PC, PROT, SOD) measured in *Scrobicularia plana* and *Mytilus galloprovincialis*, and their loadings on principal coordinate analysis (PCO1–PCO7) axes derived from the PCO analysis explaining >80% of total variation.

	AChE	ETS	GSTS	LPO	PC	PROT	SOD
PCO1 66.3%	0.97162089	0.54907359	0.95464197	-0.9571688	0.65371886	0.79551853	-0.7130379
PCO2 16.6 %	-0.1815669	0.72970772	-0.1873088	-0.1598123	-0.0299047	0.34930713	0.6405474
PCO3 9.9 %	-0.0515354	0.17276644	0.1871566	-0.0552532	-0.7508899	0.0573927	-0.2368333
PCO4 5.8 %	-0.1249081	-0.3572108	-0.1145691	-0.1276704	-0.069566	0.47801584	0.04224907
PCO5 1.2 %	0.05794794	-0.0914594	0.04521869	-0.1892424	-0.054606	-0.1137705	0.14611645
PCO6 0.1%	-0.0312497	0.0152334	-0.056037	-0.0558092	0.00855401	-0.0198711	-0.0452865
PCO7 0%	-0.0198465	-0.000386	0.01534795	-0.0028162	0.00523093	-0.0017699	-0.0001911
